# Transforming evidence-based clinical guidelines into implementable clinical decision support services: the CAREPATH study for multimorbidity management

**DOI:** 10.3389/fmed.2024.1386689

**Published:** 2024-05-27

**Authors:** Mert Gencturk, Gokce B. Laleci Erturkmen, A. Emre Akpinar, Omid Pournik, Bilal Ahmad, Theodoros N. Arvanitis, Wolfgang Schmidt-Barzynski, Tim Robbins, Ruben Alcantud Corcoles, Pedro Abizanda

**Affiliations:** ^1^SRDC Software Research & Development and Consultancy Corporation, Ankara, Türkiye; ^2^Department of Computer Engineering, Middle East Technical University, Ankara, Türkiye; ^3^Department of Electronic, Electrical and Systems Engineering, School of Engineering, University of Birmingham, Birmingham, United Kingdom; ^4^Digital & Data Driven Research Unit, University Hospitals Coventry & Warwickshire NHS Trust, Coventry, United Kingdom; ^5^University Hospital OWL, Bielefeld, Germany; ^6^Geriatrics Department, Complejo Hospitalario Universitario de Albacete, Albacete, Spain; ^7^CIBER de Fragilidad y Envejecimiento Saludable (CIBERFES), Instituto de Salud Carlos III, Madrid, Spain; ^8^Facultad de Medicina de Albacete, Universidad de Castilla-La Mancha, Albacete, Spain

**Keywords:** clinical decision support, clinical guideline, automation, integrated care, multimorbidity, dementia, HL7 FHIR

## Abstract

**Introduction:**

The CAREPATH Project aims to develop a patient-centered integrated care platform tailored to older adults with multimorbidity, including mild cognitive impairment (MCI) or mild dementia. Our goal is to empower multidisciplinary care teams to craft personalized holistic care plans while adhering to evidence-based guidelines. This necessitates the creation of clear specifications for clinical decision support (CDS) services, consolidating guidance from multiple evidence-based clinical guidelines. Thus, a co-creation approach involving both clinical and technical experts is essential.

**Methods:**

This paper outlines a robust methodology for generating implementable specifications for CDS services to automate clinical guidelines. We have established a co-creation framework to facilitate collaborative exploration of clinical guidelines between clinical experts and software engineers. We have proposed an open, repeatable, and traceable method for translating evidence-based guideline narratives into implementable specifications of CDS services. Our approach, based on international standards such as CDS-Hooks and HL7 FHIR, enhances interoperability and potential adoption of CDS services across diverse healthcare systems.

**Results:**

This methodology has been followed to create implementable specifications for 65 CDS services, automating CAREPATH consensus guideline consolidating guidance from 25 selected evidence-based guidelines. A total of 296 CDS rules have been formally defined, with input parameters defined as clinical concepts bound to FHIR resources and international code systems. Outputs include 346 well-defined CDS Cards, offering clear guidance for care plan activities and goal suggestions. These specifications have led to the implementation of 65 CDS services integrated into the CAREPATH Adaptive Integrated Care Platform.

**Discussion:**

Our methodology offers a systematic, replicable process for generating CDS specifications, ensuring consistency and reliability across implementation. By fostering collaboration between clinical expertise and technical proficiency, we enhance the quality and relevance of generated specifications. Clear traceability enables stakeholders to track the development process and ensure adherence to guideline recommendations.

## 1 Introduction

In the ever-evolving landscape of healthcare, the rising prevalence of multimorbidity combined with the complexity of medical knowledge poses significant challenges to clinical decision-making (1). Clinical guidelines, grounded in evidence-based practice, serve as essential tools for healthcare professionals in delivering optimal patient care (2). Nevertheless, the manual execution of these guidelines frequently leads to variations in practice, inefficiencies, and suboptimal outcomes, seemingly making the achievement of integrated care an overwhelming challenge (3, 4).

Integrated care is an organization-focused intervention that encompasses case-management, continuity of care, disease management, service integration, and multidisciplinary teamwork (5). It is designed to address the health and social needs of individuals living with multimorbidity, with the goal of reducing adverse healthcare outcomes, including potentially preventable hospitalizations (6). Older adults with multimorbidity can receive assistance in their own homes through Information and Communication Technologies (ICT) solutions. These solutions support them in their activities of daily living, help manage medical conditions and medications, and involve them in the healthcare process. Additionally, ICT solutions also improve physical activity and nutrition, reduce frailty, and facilitate health monitoring (7). While certain challenges remain to be addressed with these solutions, including concerns regarding data privacy and security threats, they hold significant potential for facilitating the transition from conventional medical practices to remote medicine (8, 9).

Computer-interoperable clinical guidelines play a crucial role in advancing such ICT solutions and digitizing healthcare (10). They enable the implementation of personalized clinical decision support (CDS) systems, aiding healthcare professionals in adhering to complex clinical protocols and facilitating guideline integration into daily practice. CDS systems integrate patient-specific data with evidence-based guidelines, providing real-time, personalized recommendations to healthcare providers. This integration holds great promise in streamlining clinical workflows, reducing errors, and ultimately enhancing patient outcomes (11, 12). Although CDS systems have undergone swift advancement since their initial implementation in the 1980s, their full adaptation in routine clinical practice has not yet been fully achieved for many reasons, such as ethical and legal issues, the intellectual challenge of creating knowledge, and technical dimensions of delivering CDS (13–15). Software engineers face challenges in understanding clinical guidelines due to a lack of medical expertise, which hampers their ability to automate CDS services, while clinicians without technical proficiency struggle to validate CDS implementations to ensure they align with guideline recommendations. The situation becomes more difficult when patients have multimorbidity conditions, because clinical guidelines are typically designed for individual conditions, and while they may address decision-making regarding other morbidities, they lack a systematic approach to identifying relationships between guidelines for different conditions (16).

The CAREPATH Project^1^ aims to deliver a patient-centered integrated care platform to meet the needs of older patients with multimorbidity, including mild cognitive impairment (MCI) or mild dementia (MD) (17). Dementia and MCI are two of the most debilitating chronic conditions in older adults, affecting approximately 7.3 and 20% of this population (18), respectively, and leading to high-impact healthcare needs. Integrated solutions are necessary to manage this condition, especially when other chronic conditions coexist. Notably, pharmacological and non-pharmacological treatments for diseases such as heart failure or diabetes may differ in older patients with dementia compared to the general population. Developing a patient-centered integrated care platform is challenging, as the vast majority of clinical guidelines that would inform these tools typically focus on a single condition (19).

To address this challenge, Robbins et al. (20) presented clinical requirements addressing the needs of this patient group in the form of a reference, consensus clinical guideline to be used for the CAREPATH project. The development of the guideline was undertaken by a Clinical Reference Group (CRG) formed by CAREPATH project clinical partners based in Germany, Spain, Romania, and the UK. After a review of the literature to identify suitable clinical guidelines, 52 guidelines covering a range of chronic conditions, multimorbidity, and co-morbidity were assessed for quality using the AGREE II methodology (21). Based on this, 25 final guidelines were selected for examination, approval, or disapproval, grouping, and consolidation by the project CRG through a modified Delphi process (22). The final agreed guidance and actions were collated into the master narrative consensus guideline. The CAREPATH consensus clinical guideline provides advice, information, and actions in the following areas: overarching principles of management, MCI and dementia, physical exercise, nutrition and hydration, common use of drugs, coronary artery disease, heart failure, hypertension, diabetes, chronic kidney disease, chronic obstructive pulmonary disease (COPD), stroke, sarcopenia, frailty, and caregiver support.

CAREPATH aims to deliver integrated care solutions to multi-disciplinary care teams, including health and social care providers, patients and their informal caregivers, enabling them to follow consensus guidelines in a personalized manner to create holistic care plans for older adults. The Adaptive Integrated Care Platform (AICP) is a clinician-facing application that allows healthcare professionals to review and update patient data retrieved from underlying Electronic Health Record (EHR) systems. It also enables them to assess personalized suggestions for editing the patient’s care plan, such as setting clinical goals, adding or updating interventions (e.g., medications, lab orders, referrals, and patient interventions like self-monitoring activities, diet, and exercise). AICP is supported by two important components: the Technical and Semantic Interoperability Suite (TIS/SIS), which facilitates integration with EHR systems (23), and CDS services that process consensus-based guideline rules to deliver personalized care plan suggestions. Once the care plan is created, the Patient Empowerment Platform (PEP), which was developed with the involvement of patients, informal caregivers, and healthcare professionals, provides personalized assistance and guidance to patients (24). It sends reminders about care plan goals and activities, presents educational materials to reinforce treatment adherence, and collects feedback from patients via Patient Reported Outcome Measures (PROMs) to conduct geriatric assessments. Finally, the Home and Health Monitoring Platform (H/HMP) provides environment-aware services to continuously collect real-time data for early detection of onset and changes in functioning, autonomy, and underlying cognitive and physiological functions of patients.

This paper introduces a robust methodology for generating implementable specifications of CDS services, aimed at automating clinical guidelines. Through a collaborative co-creation landscape, we enable clinical experts and software engineers to jointly examine guidelines and develop human-readable CDS specifications. Our approach addresses the challenge of translating guideline suggestions into actionable guidance, bridging the gap between clinical expertise and technical implementation. Key strengths include a repeatable process, traceability, and emphasis on human-readable specifications, ensuring accessibility and alignment with evidence-based practices. By fostering interdisciplinary collaboration, our methodology empowers teams to create CDS services that effectively automate clinical guidelines while tailoring care plans to individual patient needs. Our approach is based on international standards, namely CDS-Hooks and HL7 FHIR, targeting to enhance the interoperability and potential adoption of CDS services across diverse healthcare systems.

## 2 Method

**Figure 1:**
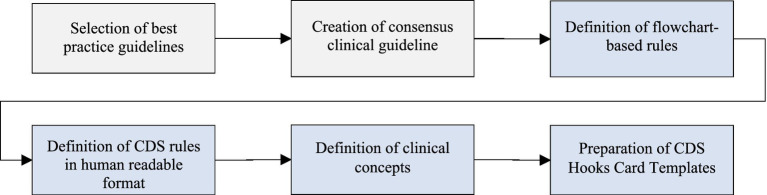
Overview of the methodology for transforming evidence-based clinical guidelines into implementable clinical decision support services.

The methodology devised to implement clinical decision support services automating evidence-based clinical guidelines consists of four steps along with the two preliminary steps as depicted in Figure 1. The selection of best practice guidelines and the creation of consensus clinical guidelines are pre-requisites of this approach. They have already been presented in (20), hence their detailed description is out of the scope of this paper. The list of the selected best practice guidelines in CAREPATH is provided in (25–49). However, it should be noted that the methodology explained in this paper can be applied to any type and number of clinical guidelines, so the selection of guidelines is not crucial for the subsequent downstream process.

In the following subsections, we explain the details of the definition of flowchart-based rules, the definition of CDS rules in human-readable format, the definition of clinical concepts, and the preparation of CDS Hooks card templates.

### 2.1 Definition of flowchart-based rules

In the first step, the consensus guideline has been converted into flowchart-based rules, allowing integration into the digital platform for delivering care to dementia patients with multimorbidity. We attempted to formulate the sentences in the consensus guideline as flowchart rules, endeavoring to identify all clinical concepts. Close cooperation between technical personnel and CRG members was carried out to clearly assess the technical feasibility and clinical effectiveness of conversion of the narrative guideline into a flowchart. We have discussed and agreed on which parts of the consensus guideline can aid the clinicians if automated as a clinical decision support service integrated into daily care practices. As a first step to create flowcharts with decision points to assess patient data, we have identified all the clinical concepts involved in textual guideline definitions. For each clinical concept, it was discussed with the CRG group whether it constitutes a diagnosis, an assessment to be conducted by the physician, a laboratory result, a medication, a clinical procedure, or a scored assessment to be performed. It was also determined whether the information would be retrieved from the EHRs of the patient, or whether it cannot be obtained from the EHR and needs assessment through physician facing CAREPATH tools, such as the AICP. Consequently, jointly agreed-upon parts of the consensus guideline have been converted into flowchart rules that pave the way for the implementation of clinical decision support services.

In our methodology, we utilized the Unified Modeling Language (UML) Activity Diagrams to draw the flowcharts. While activity diagrams are primarily used in the design phase of software engineering to describe system behavior as a workflow, researchers have also begun utilizing them for modeling clinical workflows (50–52). Activity diagrams enable us to graphically describe what clinical action needs to take place in which condition in an easy way. It also allows describing sequential and parallel processes. Activity diagrams consist of several concepts, such as activity, action, transition (control flow and object flow), decision node, swimlane and partition, each of which has a different graphical notation. In our approach, we only utilized the following concepts with the provided purpose of usage:

- Initial node: A circle representing the beginning of a workflow consisting of a set of actions or activities.
- Control flow: An arrow showing the sequence of workflow.
- Decision node: A diamond representing a test condition, such as “Has the patient met his/her blood pressure goal?.” The control flow can only continue with one of the decision paths.
- Action/activity: A (rounded) rectangle representing an action from the consensus clinical guideline such as “Consider starting monotherapy with ACE inhibitors or Angiotensin II Receptor Blockers (ARBs) or Calcium channel blockers or Thiazide diuretics by also checking possible contraindications.”
- Final node: An encircled circle representing the end of a workflow.

**Figure 2:**
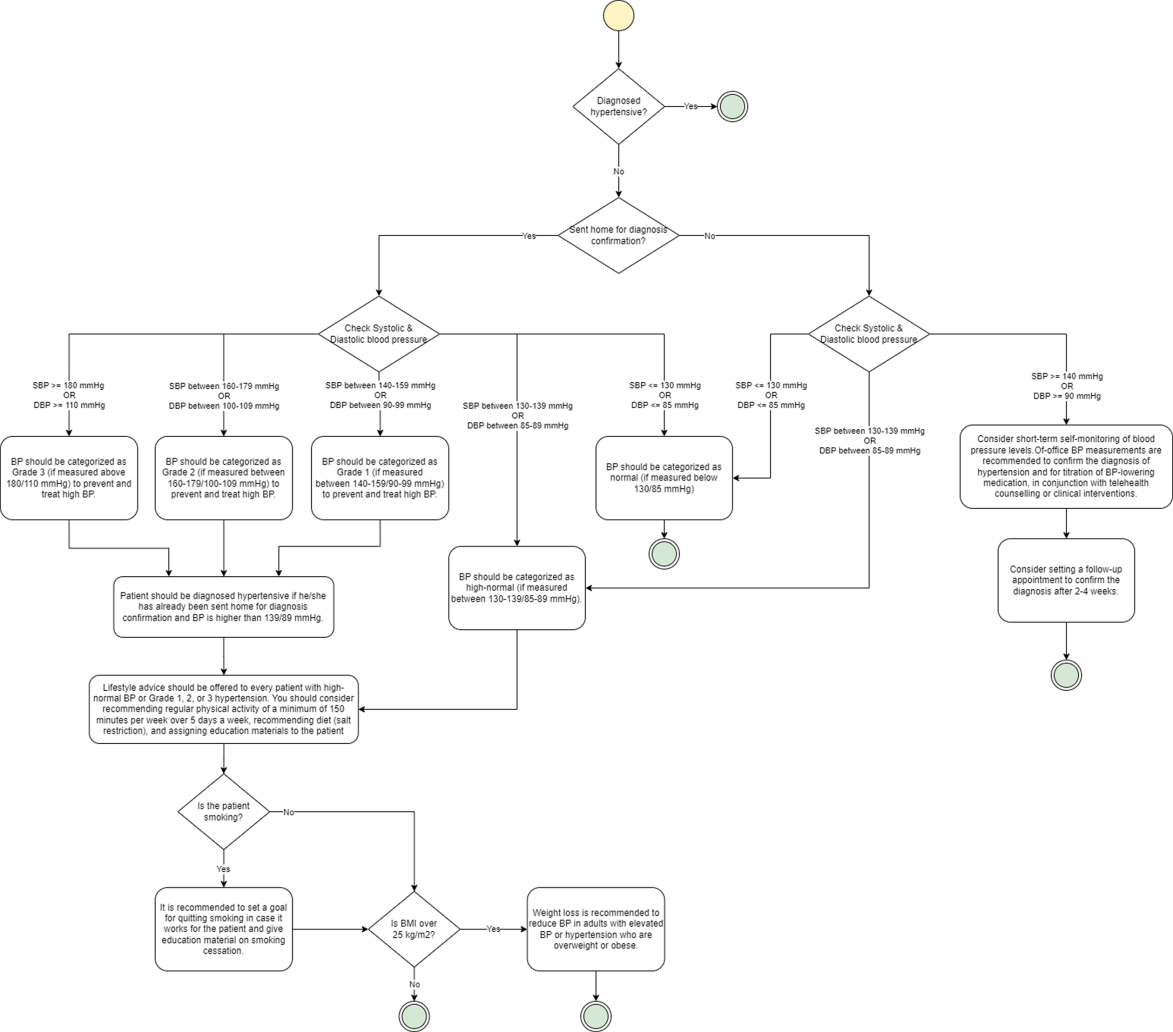
An example of a flowchart based on Hypertension guideline. The yellow circle represents the start node, while the green circles represent the end node. Diamonds are used to represent decision nodes, and rounded rectangles represent actions.

Figure 2 illustrates an example of a flowchart generated for the hypertension diagnosis procedure. If a patient has not been diagnosed hypertensive, they have not been sent home for diagnosis confirmation, and their systolic blood pressure (SBP) value is above 140 mmHg or diastolic blood pressure (DBP) value is above 90 mmHg, the guideline recommends short-term self-monitoring of blood pressure levels. It also recommends setting a follow-up appointment to confirm diagnosis after 2–4 weeks. If the SBP is between 130 and 139 mmHg or DBP is between 85 and 89 mmHg, it recommends categorizing patient’s blood pressure as high-normal. If they are below 130 mmHg or 85 mmHg, respectively, it recommends normal categorization. On the other hand, if the patient has already been sent home, then based on the SBP and DBP values, patient’s blood pressure can also be categorized as Grade 1, Grade 2, or Grade 3. In either case, the guideline recommends diagnosing the patient as hypertensive.

### 2.2 Definition of CDS rules in human readable format

In the second step, we developed directly implementable specifications for clinical decision support services to automate the consensus clinical guideline. For this purpose, we opted for the CDS Hooks formalism, which is a standard specification for clinical decision support services published by HL7.^2^ It provides an API specification enabling synchronous, workflow-triggered CDS calls that return information and suggestions. The CDS Hooks specification describes a “hook”-based pattern for invoking decision support from within a clinician’s workflow. User activity within the clinician’s workflow triggers CDS hooks in real-time. When a triggering activity occurs, the CDS Client notifies each registered CDS service for the activity. These services must then provide near-real-time feedback about the triggering event. Each service receives basic details about the clinical workflow context (via the context parameter of the hook) along with any service-specific input data required (via the pre-fetch-template parameter).

**Figure 3:**
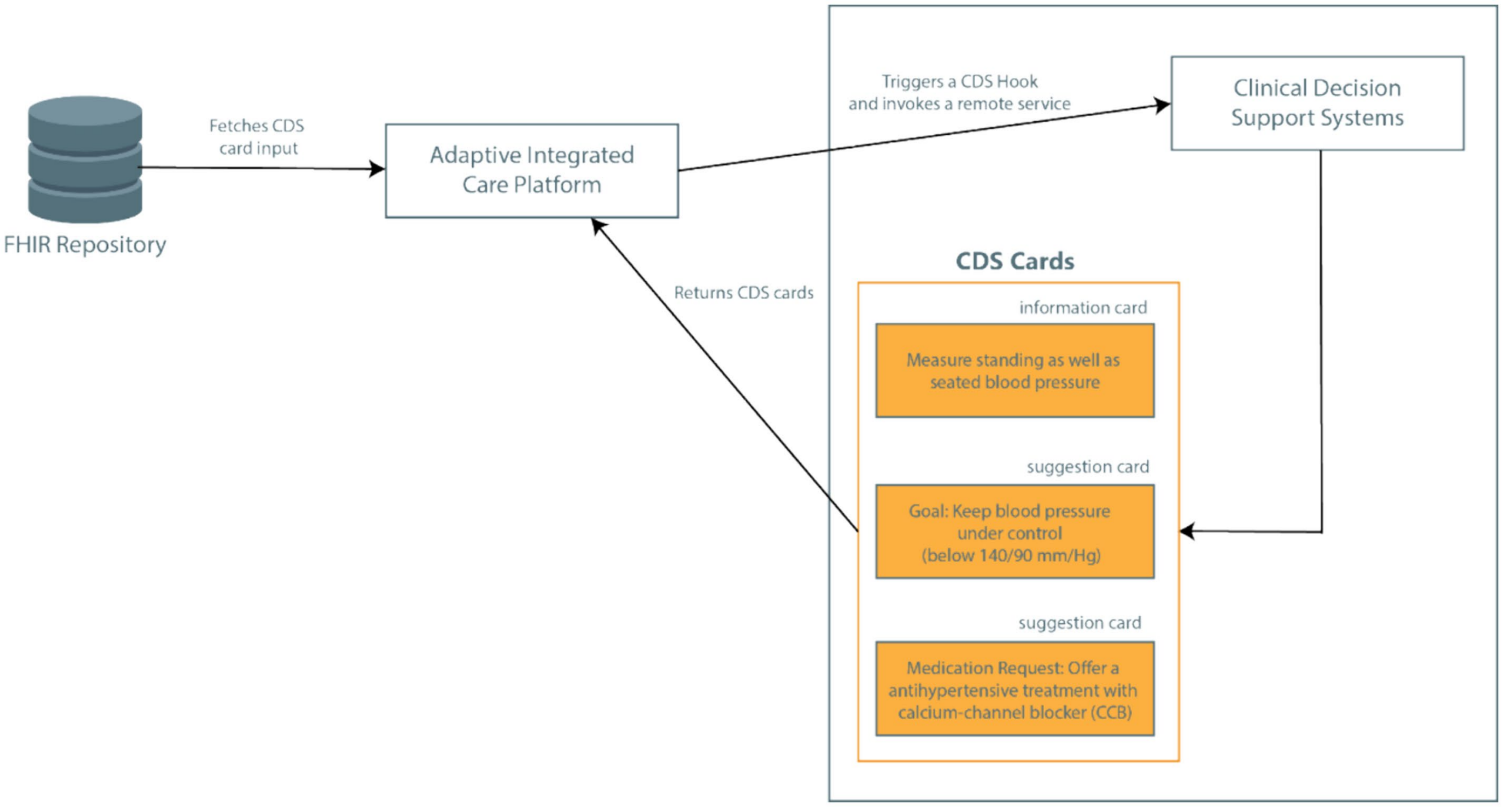
Use of CDS Hooks based services in CAREPATH architecture.

In the CAREPATH context, this mechanism is utilized as follows (see Figure 3). CDSs in CAREPATH are employed to suggest personalized goals and interventions that can be put in a care plan based on the recommendations of clinical guidelines. AICP is responsible for calling the CDS services with important patient context data, crucial for personalizing suggestions. After presenting the suggestions to clinicians via user interfaces, the care plan of the patient can be created in a guided manner.

In CAREPATH, an HL7 FHIR-based interoperability approach is followed. All components utilize HL7 FHIR as a standard-based approach to represent patient data: the patient’s EHRs retrieved from local systems by TIS/SIS are mapped to FHIR and stored in an open-source HL7 FHIR Repository, namely onFHIR.io (53), serving as a shared patient data repository. Data collected from the patient’s home via home/health monitoring devices, such as vital signs, are stored as FHIR resources by H/HMP, and patient-collected data such as symptoms are represented as FHIR resources via PEP. AICP retrieves the relevant CDS input parameters from the FHIR repository as important patient context data and passes them to CDS services. In the CDS Hooks API, the patient data collected as FHIR data is passed as input to CDS services with the ‘pre-fetch’ parameter. The CAREPATH core data model conforms to HL7 FHIR Release 4, but the implemented architecture is not bound to this specific version. It can be easily adapted to accommodate later versions or modifications.

The response of CDS services can consist of textual recommendations communicated as information cards (which can be read and assessed by the clinician to create a care plan manually, such as adding medications based on the detailed guides about possible adverse reactions) or as directly reusable care plan components communicated as suggestion cards in conformance with the CDS Hooks API. In suggestion cards, the recommended goals and activities are represented as FHIR resources (such as MedicationRequest, Goal, Appointment resources) which can be used to constitute the care plan of the patient.

Based on the CDS Hooks standard, each CDS service can return any number of cards in response to the hook. Clinicians, as users, see these cards via AICP interfaces - one or more of each type – embedded in the workflow, and can interact with them as follows:

- **Information cards** provide text for the user to read. In our methodology, guidance from clinical guidelines, which may not be feasible or practical to automate but still provide crucial information to assist clinicians in creating individualized care plans, is represented as information cards. For example, in the Hypertension guideline, the guideline recommends discussing whether the patient is taking their medication as prescribed before considering changes to drug therapy, following the National Institute for Health and Care Excellence (NICE)’s guideline on medicines adherence (54). This guidance is presented as an information card and can be viewed by clinicians in a graphical user interface (UI) for reading and acting upon it. Example representations of information cards in CAREPATH AICP are illustrated in the Results section.
- **Suggestion cards** provide a specific recommendation for which the CDS Client renders a button that the user can click to accept. Clicking automatically populates the suggested change into the clinician’s UI. In CAREPATH, CDS services can recommend adding certain care plan activities such as Referral Requests, Appointment Requests, and Lab Test Orders. These are represented as FHIR resources (as detailed in Section 2.4) and presented to the user with checkboxes via AICP. Clinicians can add them directly to the patient’s care plan by clicking on the checkboxes next to these suggestions.

The flowcharts have been reviewed together with CRG members to determine the parts of the consensus guideline that should be presented as information cards or suggestion cards, in order to create a practical tool that can be easily utilized by clinicians as a part of their daily clinical workflow.

**Figure 4:**
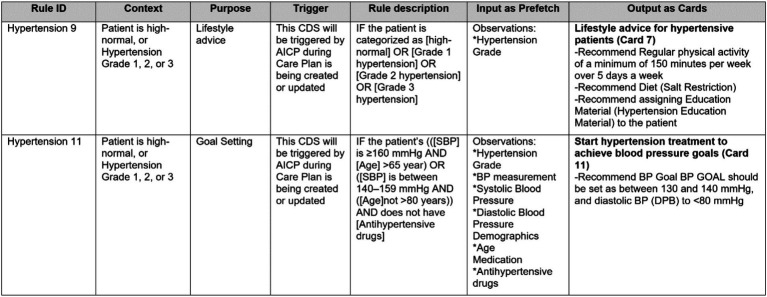
A partial view of a CDS Hooks specification table, illustrating several CDS rules from Hypertension guideline.

We created a formal template to document CDS Hooks specifications for delivering the advice, information, and actions suggested for each area addressed by the consensus clinical guideline as depicted in Figure 4.

In this table, each flowchart rule identified in the first step is represented as a row. The columns of this template can be summarized as follows:

- Each rule is identified with a unique **identifier**. We begin with the section title of the consensus guideline and assign a unique number for each rule, such as ‘Hypertension 1,’ ‘Hypertension 2,’ ‘Diabetes 1,’ ‘COPD 1’ and so on.
- Each rule has a **context** attribute, which is mostly informative and describes the current state of the patient for which the rule will be applied.
- Each rule has a purpose. The **purpose** field is critical for CDS specifications. We have examined and categorized the purpose of the advice, information, and actions suggested by the consensus guideline into the following categories:
  - Information
  - Goal management
  - Diagnosis
  - Lifestyle advice (Nutritional intervention and Physical exercise)
  - Drug treatment
  - Adverse events and medication contraindications
  - Symptom assessment
  - Complication management
  - Planning next visit

These purpose categories are utilized to group the suggestions, and separate CDS service implementations are done based on these categories. This facilitates presenting guidance from consensus guideline in a modular way in the user interfaces provided to clinicians. Different panels of the AICP pages are configured to be linked with different CDS service instances based on the purpose category, allowing clinicians to easily review the guidance provided by the consensus guideline.

- Each CDS rule has a **triggering condition**. Most of the time, for CDS rules automating clinical guideline suggestions, the triggering component is AICP. AICP calls the CDS services with the required input. Whenever the input parameters are updated from the user interface of AICP, the CDS services are triggered again.
- **Rule descriptions** are mainly retrieved from the consensus guideline and formalized to be easily converted into computer-interpretable rules. Each patient parameter represented as a clinical concept is enclosed within brackets (e.g., [SBP] designated for systolic blood pressure).
- Parameters represented as clinical concepts used in Rule descriptions within brackets are listed input parameters in the “**Input as prefetch**” column. These parameters need to be pre-fetched by the CDS client as FHIR resources from the FHIR repository and passed to the CDS services as an input. For this purpose, these parameters are first mapped to FHIR Resources, such as Condition, Observation, and Medication, and then represented as clinical concepts as explained in Section 2.3.
- Finally, the output of CDS rules is briefly described as CDS Hooks cards. Here in this table, only the titles, card numbers, and summaries are presented. As part of the CDS Hooks specification of CDS Services, there is a separate sheet where all the identified cards are clearly described, as presented in Section 2.4.

### 2.3 Definition of clinical concepts

Clear consensus on clinical concepts is a crucial step in CDS implementation for processing patient data to provide personalized suggestions. It is a step forward to create a common dictionary between clinical experts in CRG and technical experts who will implement CDS services. It is also essential to establish semantic interoperability with existing EHR systems to collect patient parameters in a machine-processable manner.

**Figure 5:**
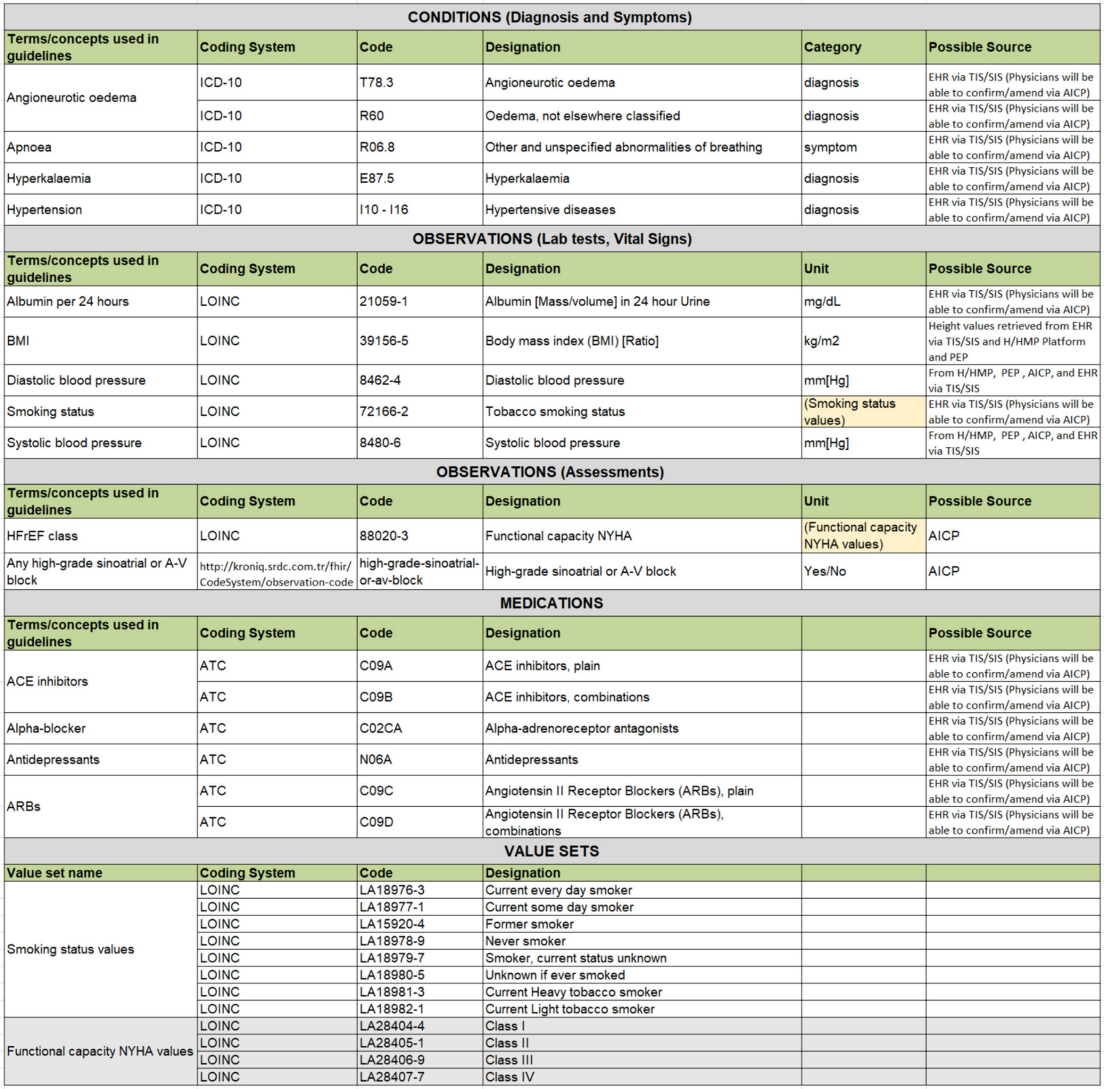
An excerpt from Hypertension clinical concepts table showcasing examples across different types.

In this step, the parameters identified in rule descriptions and the “Input as prefetch” column are represented as clinical concept definitions (see Figure 5 for examples). Firstly, as CDS Hooks specifications require CDS parameters as HL7 FHIR resources, we have categorized clinical concepts as Condition, Observation, and Medication resources.

The second important step is to bind each clinical concept to a code from international code systems. Based on discussions with CRG members, conditions have been coded either with ICD-10 or SNOMED CT codes, with categorization as diagnoses or symptoms. Medications are uniformly coded with ATC codes, while lab tests represented as FHIR Observations are coded with LOINC codes. Additionally, the agreed-upon unit of the lab test result observation is specified in reference to UCUM.

Assessments to be carried out by clinicians via AICP interfaces are also represented as FHIR Observation resources. These are coded with LOINC or SNOMED CT whenever possible. In instances where a direct mapping to a code in international code systems such as LOINC and SNOMED CT is not feasible, local codes have been created to designate these observations. The data types of these assessments, represented as FHIR Observation resources, are usually specified either as boolean Yes/No values, or as a value-set. Value-sets define a set of codes drawn from one or more code systems as possible values of these assessment observations. For example, such a value-set for representing smoking status observation is presented in Figure 5, where a set of LOINC codes is selected to represent possible values of a smoking status observation.

Additionally, the possible sources of these parameters have been identified. Some can be directly extracted from the patient’s EHR, while others require assessments during the visit, recorded via AICP. Some parameters can be retrieved from PEP, and others from H/HMP. This approach ensures that rule implementers can have a clear understanding of the clinical concepts to be processed by the CDS service implementation as parameters.

### 2.4 Preparation of CDS hooks card templates

After CDS rules are defined in a human-readable format, where the relevant clinical concepts are identified, the fourth step involves further detailing the specifications of CDS outputs identified in rule definitions. For this purpose, we have prepared CDS Hooks card templates. In successful responses, CDS Services respond with a 200 HTTP response containing an object that includes an array of cards.

**Figure 6:**
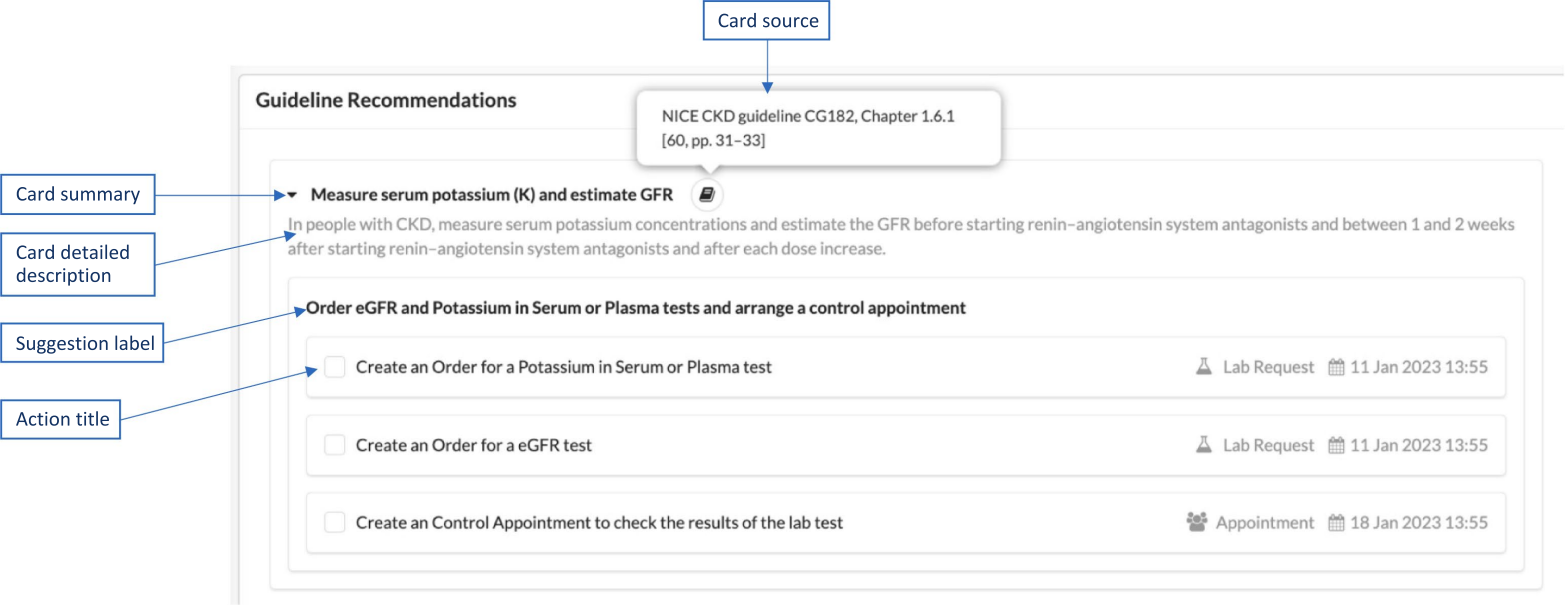
Presenting CDS Hooks cards in a user interface, such as AICP.

Each of the cards identified in the Rules template is specified with all the details required in the CDS Hooks standard specification, as explained below. An example illustration of these in a user interface, such as AICP, is displayed in Figure 6.

- **Summary**: A short (usually one sentence) explanation of the suggestion, displayed in user interfaces as the title of the card.
- **Detail**: A detailed description sourced from the consensus clinical guideline. This description is displayed when the user clicks the arrow on the right side of the card title. It can be represented as plain text or in GitHub Flavored Markdown language.^3^ This field is optional.
- **Source**: The primary source of guidance for the decision support represented by the card. In CAREPATH, we provide the exact section number and page number of the referenced clinical guideline (e.g., “Holistic patient centered CAREPATH best practice guideline, Chapter 12.2 [pp. 40]”).
- **Suggestions**: An array of suggestions that allows a service to recommend a set of changes in the context of the current activity (e.g., adjusting the dose of a currently prescribed medication for the medication-prescribe activity). Each suggestion can contain an array of Actions, each defining a suggested action. Within a suggestion, all actions are logically ANDed together, meaning that selecting a suggestion selects all the actions within it. If there are alternative suggestions, separate suggestions should be created as part of the suggestions array. Each suggestion must have a label summarizing the suggested actions.
  - Each **Action** needs to have a type, which can be “create,” “delete,” or “update.” In the CAREPATH context, “create” means that the suggested actions (such as referral, appointment, lab test request) will be added as care plan activities to the care plan; “delete” means removing an existing care plan activity from the care plan, and “update” means updating an existing care plan activity in the care plan.
  - A human-readable description of the suggested action may be presented to the end-user, along with a description of the FHIR Resource that is suggested to be created, updated, or deleted. Therefore, each Action needs to have short title summarizing the suggested action. The presentation of suggestions and their corresponding actions within AICP is depicted in Figure 6.

If an Information card is suggested by the consensus guideline, then only the first three attributes (i.e., summary, detail, and source) are necessary. In other words, Information cards do not contain any suggestions, as their purpose is only to provide some information. An example of an information card definition as a part CAREPATH CDS specifications is presented in Card 4 in Table 1.

**Table 1:** An example of an Information Card (Card 4) and a CDS card for arranging a follow-up visit to assess treatment effectiveness, containing Appointment and Lab Order actions (Card 21).

CARD 4				
Summary		Consider hypertension diagnosis with category Grade 1.		
Detailed description		BP should be categorized as normal (if measured below 130/85 mmHg), high-normal (130–139/85–89 mmHg), grade 1 (140–159/90–99 mmHg), grade 2 (160–179/100–109 mmHg) or grade 3 (≥ 180/110 mmHg) to prevent and treat high BP.		
Source		Holistic patient-centered CAREPATH best practice guideline, Chapter 12.2 [pp. 40]		
		Link to CAREPATH best practice guideline		
CARD 21				
Summary		Arrange a follow-up visit in 1 month.		
Detailed description		* Adults initiating a new or adjusted drug regimen for hypertension should have a follow-up evaluation of adherence and response to treatment at monthly intervals until control is achieved. * Renal function should be frequently assessed to detect possible increases in serum creatinine and reductions in eGFR as a result of BP-related reductions in renal perfusion.		
Source		Holistic patient centered CAREPATH best practice guideline, Chapter 12.3.1 [pp. 41 and 42]		
		Link to CAREPATH best practice guideline		
Suggestion 1				
label	Consider checking lab tests for eGFR and serum creatinine and setting a follow-up appointment within a month.			
ACTION 1	type	create		
	description	Consider setting a follow appointment after 1 month for follow-up evaluation of adherence and response to treatment.		
	resource	Appointment	extension	http://kroniq.srdc.com.tr/fhir/StructureDefinition/title | Follow appointment after 1 month for follow-up evaluation of adherence and response to treatment
			description	Follow appointment after 1 month for follow-up evaluation of adherence and response to treatment.
			status	proposed
			start	*{{Today + 1 month}}*
			specialty	–
ACTION 2	type	create		
	description	Consider ordering a serum creatinine test to assess renal function.		
	resource	Service Request	status	draft
			extension	http://kroniq.srdc.com.tr/fhir/StructureDefinition/title | Serum creatinine test
			intent	proposal
			occuranceDateTime	*{{Today + 1 month}}*
			category	http://www.kroniq.srdc.com.tr/fhir/care-plan-activity-category | lab-request | Lab Request
			code	LOINC | 2,160–0 | Creatinine [Mass/volume] in Serum or Plasma
			performerType	–
			performer	Patient
			text.status	generated
			text.div	Have serum creatinine before the control visit

Suggestion cards, on the other hand, always contain at least one Suggestion, which includes at least one Action. In CAREPATH, we have defined 7 types of actions, which are lab order, referral, appointment, patient activity, education material, goal, or medication. Within each Action, the exact FHIR Resource suggested to be added to the patient’s care plan should be present. In CAREPATH, we use the following FHIR resources to represent consensus guideline suggestions as care plan components: *ServiceRequest*, *Appointment*, *CommunicationRequest*, *Goal*, and *MedicationRequest*. The details of different types of Actions alongside the FHIR resources used in them are explained below.

- **Lab Order Suggestions**: Consensus guidelines may suggest lab orders to be requested as part of the care plan. These are represented as the *ServiceRequest* resource in HL7 FHIR. An example of a lab order suggestion action within a suggestion card is presented in Action 2 of the card in Table 1. With the ‘category’ attribute of the *ServiceRequest* resource, we identify it as a lab request, referencing our local ‘care-plan-activity-category’ value set. The specific lab test requested is specified via the ‘code’ attribute of the *ServiceRequest* resource. In the example in Table 1, Action 2 of Card 21 suggests a lab test order for ‘Creatinine [Mass/volume] in Serum or Plasma’, indicated by the ‘2160–0’ code from LOINC. Lab order categories are always indicated via a code from LOINC in CAREPATH. If there is guidance in the consensus guideline about when this lab test needs to be conducted, this is represented via the ‘occuranceDateTime’ attribute.
- **Referral Suggestions**: Consensus guidelines may suggest referrals to specialists as part of the care plan when a second opinion is needed. These are represented as *ServiceRequest* in HL7 FHIR.^4^ The ‘category’ attribute of the *ServiceRequest* resource identifies it as a referral request, referencing our local ‘care-plan-activity-category’ value set. When guidance is available in the consensus guideline, the specialty of the practitioner to whom the referral is targeted is specified via the ‘performerType’ attribute of the *ServiceRequest* resource. For example, Action 3 in Table 2 indicates a referral to a cardiologist via the ‘175651000’ code from SNOMED CT. Here, we always provide a code from the ‘performer-role’ value set defined by HL7.^5^ If there is guidance in the consensus guideline about when this referral needs to be conducted, this is represented via the ‘occuranceDateTime’ attribute.
- **Appointment Suggestions**: Consensus guidelines may suggest appointments to be scheduled as part of the care plan. These appointments can be for regular care plan review visits, to check the effects of treatments, or to discuss the results of referrals. They are represented as *Appointment* resource in HL7 FHIR.^6^ An example appointment action within a suggestion card is presented in Action 2 of the card in Table 2. The critical attributes are the appointment description and the proposed date, which is represented via the ‘start’ attribute.
- **Patient Activity Suggestions**: Consensus guidelines may suggest certain type of activities to be carried out by the patients as part of their care plans, such as physical exercises and self-measurement of vital signs. These are represented as *ServiceRequest* resources in HL7 FHIR. An example patient activity suggestion action within a suggestion card is presented in Action 1 of the card in Table 2. With the ‘category’ attribute of the *ServiceRequest* resource, we identify that it is a patient order, referencing our local ‘care-plan-activity-category’ value set. The specific activity type to be carried out is specified via the ‘code’ attribute of the *ServiceRequest* resource. In the example in Table 2, Action 1 suggests the patient to measure their blood pressure, indicated by the ‘85354–9’ code from LOINC in the ‘code’ attribute. If there is guidance in the consensus guideline about when this activity needs to be conducted, this is represented via the ‘occuranceDateTime’ attribute. In this example, the patient is asked to measure their blood pressure twice a day.
- **Education Material Suggestions**: Consensus guidelines may suggest educational materials to be assigned to the patient as a part of the care plan. These are represented as *CommunicationRequest* resources in HL7 FHIR.^7^ The payload attribute is utilized to refer to an online educational material that can be offered to the patient via the ‘payload.contentAttachment.url’ attribute. An example is presented in Action 1 and 2 of the Lifestyle Interventions card shown in Table 3.
- **Goal Suggestions**: Consensus guidelines may suggest personalized goals to be assigned to the patient as part of the care plan. For example, in the diabetes section of the consensus guideline, personalized HbA1C, blood pressure, and lipid targets are suggested based on the patient’s various parameters, such as glucose level, age, comorbidities, and recent lab test results. These goals are represented as *Goal* resources in HL7 FHIR.^8^ The objective of the goal is indicated via the ‘description.code’ attribute, referencing international code systems. In the example presented in Table 4, the code ‘135840009’ from SNOMED CT is used to specify that this is a ‘Blood Pressure monitoring’ goal. The specifics of the goal target are specified via the ‘target’ attribute, where the ‘target.measure’ attribute indicates that this is a goal for systolic blood pressure, referencing LOINC code ‘8480–6’, with the target values indicated via the ‘target.detailRange’ attributes between 130 and 140 mmHg.
- **Medication Suggestions**: Consensus guidelines may suggest adding, updating the dose, or discontinuing a medication as part of the personalized care plan for the patient. For example, in the hypertension section of the consensus guideline, if the patient cannot achieve their blood pressure goals while already on dual medication, the consensus guideline suggests considering a triple combination of ACEi/ARB, CCB, and diuretic, while also checking for possible contraindications. These medication recommendations can be represented as *MedicationRequest* resources in HL7 FHIR.^9^ In the example presented in Table 5, the first suggestion card recommends adding a beta-blocking agent. Other possible options can be added as additional alternative suggestion cards. The code “C07” from ATC is used to specify that the recommended drug is a beta-blocking agent. Possible side effects are presented as information cards, as depicted in Table 5. Considering that there could be too many options for the clinician to decide on, especially when considering possible side effects, it is also possible to represent medication recommendations as Information cards only. This enables the clinician to manually edit the medication plan via AICP after reviewing all the guidance provided. In CAREPATH, we have chosen to follow this approach to make the CDS specifications more concise.

**Table 2:** An example of a CDS card for the management of resistant hypertension, containing Referral, Appointment and Patient Activity actions.

CARD 18				
Summary		Management of resistant hypertension.		
Detailed description		The recommended treatment strategy for resistant hypertension should include appropriate lifestyle measures and treatment with optimal or best-tolerated doses of three or more drugs, which should include a diuretic, typically an ACE inhibitor or an ARB, and a CCB. Secondary causes have to be ruled out when BP recommended treatment strategy fails to lower office systolic and diastolic BP values to <140 mmHg and/or < 90 mmHg, respectively, and the inadequate control of BP is confirmed by Ambulatory BP Monitoring or home BP monitoring in patients whose adherence to therapy has been confirmed.		
Source		Holistic patient-centered CAREPATH best practice guideline, Chapter 12.3.2 [pp. 42] and Chapter 12.2 [pp. 40]		
		Link to CAREPATH best practice guideline		
Suggestion 1				
label	Consider short-term self-monitoring of blood pressure levels to confirm inadequate control of BP.			
ACTION 1	type	create		
	description	Consider short-term self-monitoring of blood pressure levels to confirm inadequate control of BP.		
	resource	Service Request	status	draft
			extension	http://kroniq.srdc.com.tr/fhir/StructureDefinition/title | Self-monitoring of BP
			intent	proposal
			occuranceTiming	“start”: *{{Today}}*, “end”: *{{Today + 2 weeks}}*, “frequency”: 2, “period”: 1, “periodUnit”: “d”
			category	http://www.kroniq.srdc.com.tr/fhir/care-plan-activity-category | patient-order | Patient Order
			authoredOn	*Automatically set to the date the CDS call is made*
			code	LOINC | 85,354–9 | Blood pressure panel
			performer	Patient
ACTION 2	type	create		
	description	Consider setting a follow appointment to confirm resistant hypertension after 2–4 weeks.		
	resource	Appointment	extension	http://kroniq.srdc.com.tr/fhir/StructureDefinition/title | Follow appointment to confirm resistant hypertension after 2–4 weeks
			description	Follow appointment to confirm resistant hypertension after 2–4 weeks.
			status	proposed
			start	*{{Today + 2 weeks}}*
			specialty	–
ACTION 3	type	create		
	description	Consider a Referral to Cardiologist for ruling out secondary causes.		
	resource	Service Request	status	draft
			extension	http://kroniq.srdc.com.tr/fhir/StructureDefinition/title | Referral to Cardiologist
			intent	proposal
			occuranceDateTime	*{{Today}}*
			category	http://www.kroniq.srdc.com.tr/fhir/care-plan-activity-category | referral | Patient referral to specialist
			authoredOn	*Automatically set to the date the CDS call is made*
			performerType	SNOMED | 175,651,000 | Cardiologist
			performer	–
			text.status	generated
			text.div	Referral to Cardiologist for ruling out secondary causes of resistant hypertension

**Table 3:** An example of a CDS card for offering lifestyle interventions for hypertensive patients, containing Communication Request actions.

CARD 7				
Summary		Offer Lifestyle interventions for hypertensive patients.		
Detailed description		Lifestyle advice should be offered to every patient with high-normal BP or Grade 1, 2, or 3 hypertension. Please check Diet Management and Exercise Planning pages for detailed diet and exercise plans to be added to the care plan of the patient.		
Source		Holistic patient centered CAREPATH best practice guideline, Chapter 12.3.1 [pp. 41]		
		Link to CAREPATH best practice guideline		
Suggestion 1				
label	Offer lifestyle advice and educational materials to hypertensive patients for healthy diet and physical activity.			
ACTION 1	type	create		
	description	Give education material on healthy diet.		
	resource	Communication Request	status	draft
			extension	http://kroniq.srdc.com.tr/fhir/StructureDefinition/title | Education material on healthy diet
			subject	Patient
			authoredOn	*Automatically set to the date the CDS call is made*
			payload.contentAttachment.language	en
			payload.contentAttachment.url	https://www.nhsinform.scot/healthy-living/food-and-nutrition
			payload.contentAttachment.title	Diet and nutrition - benefits of a balanced diet
ACTION 2	type	create		
	description	Give education material on physical activity for healthy living.		
	resource	Communication Request	status	draft
			extension	http://kroniq.srdc.com.tr/fhir/StructureDefinition/title | Education material on physical activity for healthy living
			subject	Patient
			authoredOn	*Automatically set to the date the CDS call is made*
			payload.contentAttachment.language	en
			payload.contentAttachment.url	https://www.nhsinform.scot/healthy-living/keeping-active
			payload.contentAttachment.title	Physical activity – health benefits of exercise

**Table 4:** An example of a Goal suggestion CDS card.

CARD 11				
Summary		Systolic BP should be targeted to between 130 and 140 mmHg, and diastolic BP to <80 mmHg.		
Detailed description		The evidence supports the recommendation that multi-morbid older patients with cognitive impairment (>65 years, including patients over 80 years) should be offered BP-lowering treatment if their systolic BP is ≥160 mmHg. There is also justification to now recommend BP-lowering treatment for old patients (aged >65 but not >80 years) at a lower BP (i.e., grade 1 hypertension where systolic BP is between 140 and 159 mmHg). Systolic BP should be targeted to between 130 and 140 mmHg, and diastolic BP to <80 mmHg.		
Source		Holistic patient centered CAREPATH best practice guideline, Chapter 12.1 [pp. 40]		
		Link to CAREPATH best practice guideline		
Suggestion 1				
label	Keep blood pressure under control.			
ACTION 1	type	create		
	description	Keep systolic blood pressure under control (between 130 and 140 mm/Hg)		
	resource	Goal	lifecycleStatus	proposed
			meta.tag	http://kroniq.srdc.com.tr/fhir/CodeSystem/concept-id | GoalSystolicBP
			extension	http://kroniq.srdc.com.tr/fhir/StructureDefinition/title | Keep systolic blood pressure under control
			category	http://terminology.hl7.org/CodeSystem/goal-category | safety
			startDate	*Automatically set to the date the CDS call is made*
			description.text	Keep systolic blood pressure under control (between 130–140 mm/Hg)
			description.code	SNOMED | 135,840,009 | Blood Pressure monitoring (regime/therapy)
			target.dueDate	*{{Today + 3 months}}*
			target.measure	LOINC | 8,480–6 | Systolic blood pressure
			target.detailRange	low:130, high:140
ACTION 2	type	create		
	description	Keep diastolic blood pressure under control (below 80 mm/Hg)		
	resource	Goal	lifecycleStatus	proposed
			meta.tag	http://kroniq.srdc.com.tr/fhir/CodeSystem/concept-id | GoalDiastolicBP
			extension	http://kroniq.srdc.com.tr/fhir/StructureDefinition/title | Keep diastolic blood pressure under control
			category	http://terminology.hl7.org/CodeSystem/goal-category | safety
			startDate	*Automatically set to the date the CDS call is made*
			description.text	Keep diastolic blood pressure under control (below 80 mm/Hg)
			description.code	SNOMED | 135,840,009 | Blood Pressure monitoring (regime/therapy)
			target.dueDate	*{{Today + 3 months}}*
			target.measure	LOINC | 8,482–4 | Diastolic blood pressure
			target.detailRange	low:-, high:80

**Table 5:** An example of a Medication suggestion CDS Card (Card 31) and a possible side effect CDS card (Card 38).

CARD 31				
Summary		Consider triple combination of ACEi/ARB, beta-blocker, CCB and diuretic by also checking possible contraindications.		
Detailed description		For CAD patients who do not meet their BP goals on dual therapy, consider triple combination of ACEi/ARB, beta-blocker, CCB and diuretic by also checking possible contraindications.		
Source		Holistic patient-centered CAREPATH best practice guideline, Chapter 12.3.2 [pp. 42]		
		Link to CAREPATH best practice guideline		
Suggestion 1				
label	Consider adding Beta Blockers as a third therapy.			
ACTION 1	type	create		
	description	Consider prescribing Beta Blockers.		
	resource	MedicationRequest	lifecycleStatus	proposed
			description.text	Prescribe Beta Blocker as a part of triple therapy
			Medication.code	ATC | C07 | Beta Blocking Agents
CARD 38				
Summary		Compelling side effects for Beta-Blockers.		
Detailed description		Beta-blockers has compelling side effects for the patients with one of the following conditions: asthma or any high-grade sinoatrial or A-V block or bradycardia (heart rate < 60 beats per min).		
Source		Holistic patient centered CAREPATH best practice guideline, Chapter 12.3.2, Table 2		
		Link to CAREPATH best practice guideline		

## 3 Results

### 3.1 Output CDS rules and CDS hooks cards

Following the presented methodology, we analyzed the CAREPATH consensus clinical guideline, which provides advice, information, and actions in the following areas: overarching principles of management, mild cognitive impairment and dementia, physical exercise, nutrition and hydration, common use of drugs, coronary artery disease, heart failure, hypertension, diabetes, chronic kidney disease, COPD, stroke, sarcopenia, frailty, and caregiver support. We drew flowcharts, defined CDS rules and clinical concepts, and finally produced detailed implementable CDS-Hooks specifications for CDS services automating the following sections:

- Recommendations for the management of Mild dementia and mild cognitive impairment
- Recommendations for the management of Physical exercise
- Recommendations for the management of Nutrition and hydration
- Recommendations for the management of Commonly used drugs
- Recommendations for the management of Coronary artery disease
- Recommendations for the management of Heart failure
- Recommendations for the management of Hypertension
- Recommendations for the management of Diabetes
- Recommendations for the management of Chronic kidney disease
- Recommendations for the management of Chronic obstructive pulmonary disease
- Recommendations for the management of Stroke
- Recommendations for the management of Sarcopenia and frailty
- Recommendations for the management of Caregiver support

The full specifications are provided in the Supplementary material. In Tables 6, 7, we summarize the results of this process. The rules have been categorized under the following nine categories based on the purpose of recommendations:

1. **Diagnosis**: Guideline recommendations for diagnosing a patient’s condition based on their current health parameters and status. For instance, hypertension guidelines recommend diagnosing hypertension if the patient has already undergone home diagnosis confirmation and their blood pressure remains higher than 139/89 mmHg.
2. **Lifestyle advice**: Guideline recommendations related to nutritional intervention, physical exercise, and smoking cessation.
3. **Goal management**: Guideline recommendations for assigning patients targets to achieve, such as maintaining systolic blood pressure between 130 and 140 mmHg or providing weight loss advice to adults with elevated blood pressure or hypertension who are overweight or obese.
4. **Drug treatment**: Guideline recommendations for initiating new medication therapy for newly diagnosed patients or adjusting existing medication therapy if disease progression is not controlled.
5. **Adverse events and medication contraindications**: Guideline recommendations for informing clinicians about possible adverse events and contraindications before starting a new medication therapy. For instance, the CAREPATH consensus clinical guideline recommends closely monitoring the impact of BP-lowering drugs on the well-being of the patient due to increased risk of adverse events (e.g., injurious falls) in older adults. When combination therapy is used, it suggests starting at the lowest available doses.
6. **Information and guidance about disease management**: Includes guidance for clinicians on important aspects of disease treatment associated with cognitive impairment and dementia, reminders about assessments needed before treatment planning and presenting useful information for sharing/discussion with patients and their caregivers. For example, “Before initiating pharmacological treatment for diabetes, the person’s cardiovascular status and risk should be assessed to determine whether they have chronic heart failure” or “Keeping the environment at home safe to reduce the risk of falling and injury.”
7. **Symptom recording**: For reminding clinicians to assess patient’s specific symptoms at certain times or under certain conditions. For example, diabetes guidelines recommend assessing symptoms such as distress, disabilities, depression, anxiety, disordered eating, visual and hearing impairments, cognitive capacities, and other geriatric syndromes using a Comprehensive Geriatric Assessment at the initial visit, at periodic intervals, and when there is a change in disease, treatment, or life circumstance, including caregivers and family members in this assessment.
8. **Complication management and referrals**: Recommendations for referring patients to other departments or specialists in case of suspected complications, emergencies, or when consultancy/expertise from another specialty is required. For example, in hypertension treatment, referral to a cardiologist is recommended to rule out secondary causes if recommended treatment strategies fail to lower blood pressure values. Additionally, referral to a respiratory disease specialist is recommended for diagnosing obstructive sleep apnea if the patient exhibits symptoms such as snoring, apnea, nocturia, nocturnal dyspnea, nighttime cardiovascular events, or resistant hypertension, along with daytime sleepiness. Moreover, a referral to emergency services is advised if the patient’s clinic blood pressure exceeds 180/110 mmHg.
9. **Planning next visit**: For scheduling follow-up appointments to evaluate patient’s adherence to care plan activities and their response to treatment.

**Table 6:** The number of rules defined for different categories in each section.

	HT	DM	COPD	MD& MCI	STR	S&F	CAD	HF	CKD	CUD	N&H	PE	CS
Diagnosis	12	3	9	3	1	1	3	4	4	–	–	–	–
Lifestyle advice	3	3	–	1	–	2	2	–	1	–	11	5	1
Goal management	2	5	–	–	1	–	–	–	1	–	–	–	–
Drug treatment	17	22	12	8	7	–	9	13	20	9	–	–	–
Adverse events and medication contraindications	17	–	–	4	–	–	–	–	–	–	–	–	–
Information and guidance about management	–	9	2	19	2	2	3	1	8	–	–	–	–
Symptom recording	–	1	1	–	–	–	–	–	–	–	–	–	–
Complication management and referrals	1	10	6	1	2	2	1	1	3	–	–	–	–
Planning next visit	–	2	–	2	–	–	–	–	1	–	–	–	–
**TOTAL**	**52**	**55**	**30**	**38**	**13**	**7**	**18**	**19**	**38**	**9**	**11**	**5**	**1**

The abbreviations used in the header refer to the following: HT, Hypertension; DM, Diabetes; COPD, Chronic Obstructive Pulmonary Disease; MD&MCI, Mild Dementia & Mild Cognitive Impairment; STR, Stroke; S&F, Sarcopenia & Frailty; CAD, Coronary Artery Disease; HF, Heart Failure; CKD, Chronic Kidney Disease; CUD, Commonly Used Drugs; N&H, Nutrition & Hydration; PE, Physical Exercise; CS, Caregiver Support.

**Table 7:** The number of CDS Hooks cards defined for each section and the number of actions per type in the cards.

	HT	DM	COPD	MD& MCI	STR	S&F	CAD	HF	CKD	CUD	N&H	PE	CS
Cards	55	55	40	38	13	7	18	31	41	9	11	6	2
Information & Medication contraindication	33	21	12	27	3	4	3	6	11	1	9	5	2
Patient activity	2	1	–	–	–	–	1	–	–	–	–	1	–
Appointment	2	2	1	2	–	–	–	3	4	–	–	–	–
Referral	4	7	7	1	3	2	5	5	3	–	2	–	–
Education material	3	1	2	1	–	–	1	1	1	–	–	–	–
Goal	6	17	–	–	1	–	1	–	1	–	–	–	–
Lab request	4	6	3	3	–	–		10	17	–	–	–	–
Medication request	17	20	11	6	7	1	9	10	14	8	–	–	–
Autofill	4	–	8	–	–	–	–	6	3	–	–	–	–

Table 6 presents the number of rules defined for each section of the holistic guideline based on these categories. In total, 296 CDS rules have been defined. Among them, 117 (40%) are related to drug treatment, 46 (16%) to information and guidance about management, 40 (13%) to diagnosis, 29 (10%) to lifestyle advice, and 64 (21%) to other categories. No rules have been defined for drug treatment in the Sarcopenia & frailty, Nutrition & hydration, Physical exercise, and Caregiver support sections, because these guidelines do not directly address the treatment of specific diseases. Similarly, no rules related to diagnosis, complication management, and referral exist in the Commonly used drugs, Nutrition & hydration, Physical exercise, and Caregiver support sections. In goal management, guidelines for Hypertension, Diabetes, Stroke and Chronic kidney disease recommend setting targets for systolic blood pressure, diastolic blood pressure, weight, HbA1c, fasting glucose, LDL cholesterol, HDL cholesterol, Total cholesterol, and Hemoglobin. Since the CAREPATH study mainly focuses on multimorbidity management in the elderly with dementia, the largest number of rules for information and guidance about management has been defined in the Mild dementia & mild cognitive impairment section.

In each CDS rule, there exists one or more CDS Hooks card to achieve the specific objective of that rule. Table 7 shows the number of CDS Hooks cards defined for each section and the number of actions in those cards per action type. In the CAREPATH study, we defined 326 CDS Hooks cards to implement 296 CDS rules. Among them, the majority of the cards (191 out of 326, 59%) appeared in the Hypertension, Diabetes, COPD, and Chronic kidney disease sections, followed by 38 (12%) in MD & MCI and 31 (10%) in the Heart failure sections.

As explained in the Methodology section, a CDS Hooks card can be an information card, meaning that there is no action in it, or it can contain suggestions in which there exists at least one action. In Table 7, the number of information cards in each section is presented in the “Information & Medication contraindication” row. In hypertension, there exist 17 medication contraindication rules, which are modeled as information cards in CAREPATH. The rest of the rows in the table show the number of actions per type in the other CDS Hooks cards. Here, there is an additional type, autofill, which has not been explained in the methodology. In CAREPATH, autofill CDS Hooks cards are intended to present guideline recommendations suggesting diagnosis or assessment of a patient based on a recent measurement. For instance, hypertension guidelines recommend diagnosing Bradycardia if the patient’s heart rate is less than 60 bpm, diagnosing Hyperkaliemia if the patient’s potassium level is more than 5.5 mmol/L or considering severe left ventricular dysfunction if the patient’s left ventricular ejection fraction is less than 40%.

### 3.2 Implementation of CDS engine

In CAREPATH, based on the CDS Service specifications presented in Section 2, software engineers have implemented the CDS services via a CDS Engine implementation in the Scala programming language. For each category in each section presented in Table 6, a CDS-Hooks-complaint REST endpoint has been implemented. For some categories that contain a considerable number of rules, such as drug treatment or information and guidance about management, multiple endpoints have been created. Consequently, a total of 65 CDS-Hooks-compliant REST endpoints have been implemented.

In CAREPATH, the patient data retrieved from EHRs, created via AICP, and collected from patients via H/HMP and PEP, are all represented in HL7 FHIR and maintained in a FHIR repository. Within the implementation of CDS-Hooks endpoints in Scala, the prefetch parameters have been expressed as FHIR queries, to retrieve the indicated patient input from a FHIR server, acting as the patient data store.

The CDS Hooks cards, represented as separate tables in the CDS specifications, have been defined as parametrized JSON files, using a template language, namely Mustache. These are instantiated for each patient by filling in the placeholders with patient-specific parameters by our CDS Engine. The CDS Logic, defined as rules in the CDS specifications, is implemented as rules defined via FHIR Path expressions, mapping retrieved input parameters to pre-defined CDS Hooks Template cards. The defined CDS Hooks cards and service definitions are available as open-source on GitHub^10^.

In CAREPATH, we have preferred a Scala-based implementation. However, given the open specifications presented in Section 2, Supplementary material, and CDS-Hooks standard specifications, any other programming language could have been used to realize the implementation of these RESTful CDS services. Clear, open specifications mapping the clinical concepts to FHIR resources and international code systems, and rules defined based on these clinical concepts, enabled engineers who do not have clinical expertise to easily realize CDS implementations.

### 3.3 Usage of CDSs in a real-word environment

The Adaptive Integrated Care Platform (AICP) is one of the core components of the CAREPATH system, facilitating collaborative management of the care of multimorbid patients with mild dementia. It serves as the direct interface to care team members, allowing for the definition, updating, reconciling, and sharing of care plans, as well as the utilization of clinical decision support modules supporting these operations. It provides healthcare professionals with relevant information to guide decisions in an effective way, both during follow-up visits and in initial diagnosis processes. AICP has been implemented as a Web application providing an easy-to-navigate dashboard for care team members to view the basic medical history of the patient along with the care plan lifecycle history. The AICP care plan management graphical user interfaces have been designed to integrate the CDS services and to present the suggestions coming from CDS services in the best possible manner to facilitate care plan editing in the guidance of evidence-based clinical guidelines. The design was made with the involvement of healthcare professionals. First, the user requirements were collected through interviews conducted with them. Then, based on the user requirements, several mockups were drawn. These mockups were presented to healthcare professionals and their feedback was received. At the end of a few rounds, the final design emerged.

The input parameters of CDS services may be retrieved from the EHRs of the patient, including the patient’s existing diagnosis, medications, and lab test results, from PEP for symptoms recorded, and from H/HMP for measurements retrieved from health devices. During the analysis of CDS services, we realized that some input parameters are clinical assessments which need to be carried out by the clinician during the visit with the patient. An example could be assessing “whether the patient’s condition is stable or not.”

AICP has been designed to provide a specific page for the management of each section, described in Section 3.1; e.g., Hypertension diagnosis/treatment, Diabetes diagnosis/treatment, CAD management etc., along with additional pages to support some common functionalities such as reviewing the current status of the patient (such as physical examination, review of lab results), providing overarching lifestyle and physical exercise recommendations, and reviewing the questionnaires assigned to the patient.

**Figure 7:**
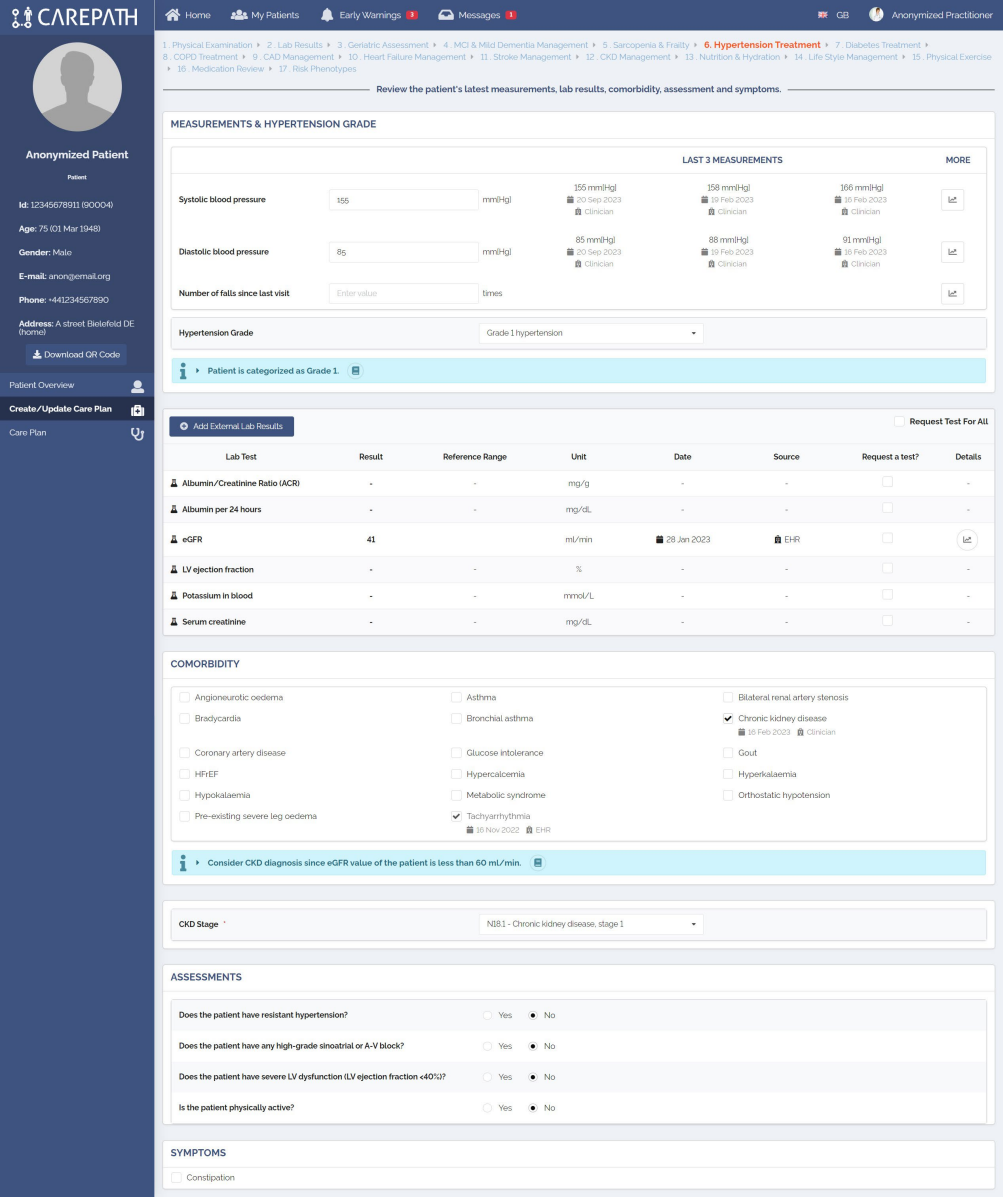
An example representation of clinical concepts identified during the definition of CDS rules in AICP pages.

**Figure 8:**
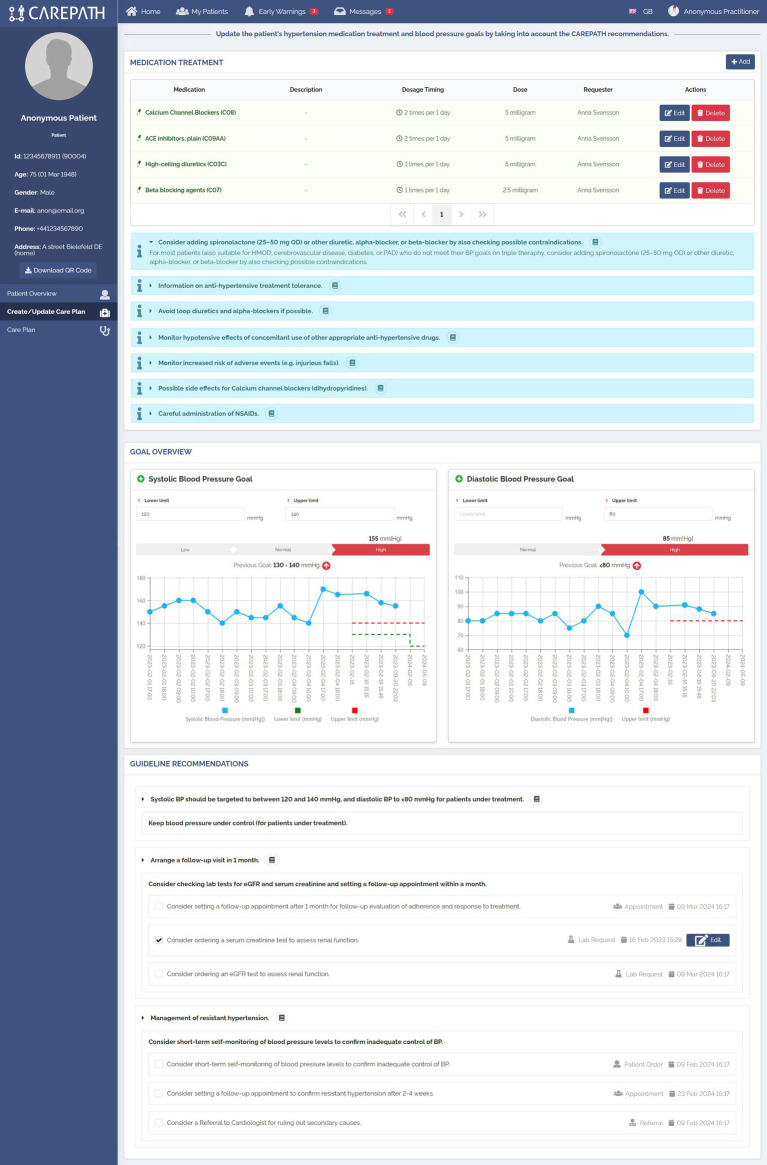
An example representation of CDS Hooks Cards in AICP interfaces.

The care plan management pages have been divided into two main parts, as illustrated in Figures 7, 8. In Part A, the clinician is reminded about the important parameters that will affect personalized decisions about care plan goals and activity suggestions. These parameters have been identified in the third step of our methodology, which is the definition of clinical concepts. The values of these concepts are mostly retrieved from EHRs, and clinicians can amend them if necessary (e.g., manually adding new lab results). Clinicians can make new assessments, mostly for assessments that need to be carried out during that encounter. Figure 7 shows the first part of the Hypertension treatment page consisting of six different panels. In the first panel, the clinician examines the patient’s latest systolic and diastolic blood pressure measurements as well as the number of falls since the last visit. The clinician can also record new values for those fields. Based on the latest systolic and diastolic blood pressure values, the guideline recommends categorizing the patient as Grade 1. In the second panel, the lab results of the patient are presented. It should be noted that these panels do not present the full medical summary of the patient. For each section, such as hypertension management, only the lab results, conditions, symptoms, assessments, etc. that are necessary for clinical assessment in the context of this section (that are listed in the clinical concepts table of the respective CDS services) are presented. In the third panel, comorbidities are shown. In the example, the CDS services automating hypertension guideline recommended CKD diagnosis, because the patient’s eGFR value is less than 60 mL/min. In the fourth and fifth panels, assessments and symptoms are presented, respectively.

Based on the reviewed patient data and the provided clinical assessments in Part A, CDS services run in the background and provide personalized suggestions about what needs to be put in the care plan of the patient in Part B, such as goals (e.g., personalized systolic blood pressure, LDL cholesterol, HbA1c target), control appointments, lab test requests, referrals, medication requests, education materials, and patient orders (e.g., measuring blood pressure at home).

Figure 8 displays the implementation of Part B in the Hypertension treatment page, consisting of three panels. As explained in Section 2.4, in CAREPATH, we have chosen to represent medication recommendations as Information cards and enable the clinician to manually edit the medication plan. Therefore, in the Medication treatment panel, the medication-related guideline recommendations are presented under the medication list, and the clinician is provided with add, edit, and delete buttons to update the patient’s medication treatment plan.

In the Goal Overview panel, the clinician can see the most recent systolic and diastolic blood pressure measurements of the patient in a chart view, observe the patient’s adherence to the previous goals, and update the goals based on the guideline recommendations.

The guideline recommendations, selected by the CDS Engine based on the patient parameters provided in Part A, are presented at the end of the page. Clinicians can decide whether to add a suggested item to the care plan or not by clicking on the checkbox near it. If needed, they can edit their details (e.g., the date of a control appointment). In the example shown in Figure 8, the guideline recommended targeting systolic BP between 120 and 140 mmHg and diastolic BP below 80 mmHg for the patient who is under treatment. It also recommended arranging a follow-up visit in 1 month and ordering lab tests for eGFR and serum creatinine. Since the patient has resistant hypertension (because the patient did not meet his BP targets on triple therapy), the guideline also recommended short-term self-monitoring of blood pressure levels to confirm inadequate control of BP, setting a follow-up appointment to confirm resistant hypertension after 2–4 weeks, and a referral to cardiologist for ruling out secondary causes.

## 4 Discussion

This paper presents a methodology for generating implementable specifications for clinical decision support (CDS) services aimed at automating clinical guidelines. We have established a co-creation framework facilitating collaborative exploration of clinical guidelines by both clinical experts and software engineers. Through a systematic, traceable approach, our methodology enables the generation of open, human-readable CDS specifications. This open and traceable co-creation approach has especially helped us to address the challenges of automating multimorbidity guidelines. We have demonstrated that it is technically possible to consolidate suggestions from multiple conflicting guidelines and transform them into implementable specifications. We believe this methodology contributes to making healthcare more manageable for healthcare providers dealing with multiple chronic conditions and provides a practical example for future studies.

Understanding clinical guidelines poses a significant challenge for software engineers lacking medical expertise, hindering their ability to develop CDS services for automation (55). Conversely, clinicians without technical proficiency encounter difficulties in validating CDS implementations to ensure alignment with guideline recommendations. Our approach addresses these challenges by fostering interdisciplinary collaboration, allowing both groups to collectively translate clinical guideline suggestions into actionable directives for personalized care plan development.

Key strengths of our methodology include:

- Repeatable Process: Our methodology offers a systematic, replicable process for generating CDS specifications, ensuring consistency and reliability across implementations.
- Co-Creation Landscape: By establishing a collaborative environment, we facilitate synergy between clinical expertise and technical proficiency, enhancing the quality and relevance of generated specifications.
- Traceability: Our approach provides clear traceability, enabling stakeholders to track the development process and ensure adherence to guideline recommendations.
- Human-Readable Specifications: We emphasize the creation of human-readable specifications, enhancing accessibility and facilitating comprehension for stakeholders across disciplines.
- Actionable Guidance: Our methodology translates clinical guideline suggestions into actionable guidance, enabling the creation of personalized care plans tailored to individual patient needs.

By bridging the gap between clinical expertise and technical implementation, our methodology empowers interdisciplinary teams to develop CDS services that effectively automate clinical guidelines while ensuring alignment with evidence-based practices.

We have adopted a standardized approach guided by CDS Hooks Specifications, leveraging HL7 FHIR to define clinical concepts. Our methodology ensures clarity by precisely delineating the input/output parameters of CDS services in alignment with HL7 FHIR Resources, grounding clinical semantics within international code systems. This establishes a universal, shared lexicon—facilitating seamless communication between clinical and technical experts. Moreover, our clear specifications streamline the implementation of CDS services, as input parameters can be readily accessed from a FHIR repository via FHIR queries. By adhering to standards and facilitating easy mapping to FHIR-based implementations, our research enhances the interoperability and potential adoption of CDS services across diverse healthcare systems. This robust framework not only accelerates integration with external health IT systems but also paves the way for widespread implementation, thereby maximizing the impact of our research in clinical practice. In doing so, it complements prior studies facing challenges in disseminating and sharing knowledge artifacts for clinical decision support across different electronic health record platforms (56, 57).

CDS services for multimorbid older adults with MCI/MD need to address “whole-of-person” interventions to improve their quality of life (19), considering not only social issues but also physical and psychological difficulties (58). The CDS services implemented, following the methodology outlined in this paper, take a holistic approach to these patients, including specific healthcare conditions not typically found in guidelines, such as nutrition, exercise, frailty, and sarcopenia. Furthermore, they enable the entire healthcare team to participate in the care process using the same platform, considering not only patients’ diseases but also environmental factors, caregiver support, quality of life, and psychosocial conditions.

The importance of patient privacy and data security in healthcare delivery necessitates careful planning and robust protection measures, particularly in highly automated workflows (59). Although the methodology outlined in this paper allows for the automation of clinical guidelines by producing implementable specifications for CDS services, it is limited to semi-automation, hence it does not provide a methodology for full automation. Healthcare professionals are still required to review CDS recommendations, make decisions, and exercise judgment at critical decision points in the workflow.

In future work, the usability, safety and technology acceptance of the CAREPATH ICT platform, including the developed tools and implemented CDS services, will be evaluated in a Technical Validation and Usability (TVU) study. This study will involve 16 patients with their informal caregivers and 16 healthcare professionals. Additionally, a Clinical Investigation (CI) involving over 200 patients will be conducted. These evaluations will take place in four European countries (Spain, Romania, Germany and the United Kingdom) over a period of 2 years.

## Data availability statement

The original contributions presented in the study are included in the article/Supplementary material, further inquiries can be directed to the corresponding author.

## Author contributions

MG: Conceptualization, Methodology, Software, Writing – original draft. GL: Conceptualization, Methodology, Project administration, Software, Writing – original draft. AA: Software, Writing – original draft. OP: Software, Writing – review & editing. BA: Validation, Writing – review & editing. TA: Validation, Writing – review & editing. WS-B: Formal analysis, Investigation, Resources, Writing – review & editing. TR: Formal analysis, Investigation, Resources, Writing – review & editing. RA: Formal analysis, Investigation, Resources, Writing – review & editing. PA: Formal analysis, Investigation, Resources, Writing – review & editing.

## Footnotes

^1^CAREPATH Project Website, https://www.carepath.care/.

^2^CDS Hooks Specifications, https://cds-hooks.hl7.org/.

^3^GitHub Flavored Markdown Spec, https://github.github.com/gfm/.

^4^HL7 FHIR, Release 4, ServiceRequest Resource, http://hl7.org/fhir/r4/servicerequest.html.

^5^HL7 FHIR, Release 4, Performer Role Value Set, https://build.fhir.org/valueset-performer-role.html.

^6^HL7 FHIR, Release 4, Appointment Resource, http://hl7.org/fhir/r4/appointment.html.

^7^HL7 FHIR, Release 4, Communication Request Resource, http://hl7.org/fhir/r4/communicationrequest.html.

^8^HL7 FHIR, Release 4, Goal Resource, http://hl7.org/fhir/r4/goal.html.

^9^HL7 FHIR, Release 4, Medication Request Resource, https://hl7.org/fhir/R4/medicationrequest.html.

^10^CAREPATH CDS Specifications, https://github.com/srdc/carepath-cds-specifications.

## Supplementary material

- CAD_CDSHooksSpecifications.xlsx
- CareGiverSupport_CDSHooksSpecifications.xlsx
- CKD_CDSHooksSpecifications.xlsx
- CommonlyUsedDrugs_CDSHooksSpecifications.xlsx
- COPD_CDSHooksSpecifications.xlsx
- Diabetes_CDSHooksSpecifications.xlsx
- HF_CDSHooksSpecifications.xlsx
- Hypertension_CDSHooksSpecifications.xlsx
- MCI_CDSHooksSpecifications.xlsx
- NH_CDSHooksSpecifications.xlsx
- PhysicalExercise_CDSHooksSpecifications.xlsx
- SarcopeniaFrailty_CDSHooksSpecifications.xlsx
- Stroke_CDSHooksSpecifications.xlsx

Additionally, the custom Value Set for listing care plan activity categories is provided in the following document.

- ValueSet_CarePlanActivityCategories.xlsx
